# Supplementary material for: Cognitive impairment and reporting of hypertension among adults in india: Evidence from a population-based study
Source: PLOS Ment Health. 2025 May 12;2(5):e0000003. doi: 10.1371/journal.pmen.0000003 (PMC12798364; doi:10.1371/journal.pmen.0000003)
Supplement: S1 Table — (DOCX) [file pmen.0000003.s001.docx]

**A description of domain-wise cognitive measures**

| **Domain** | **Measure** | **Measurement** | **Range** |
| --- | --- | --- | --- |
| **Memory** | Immediate word recall | Interviewer read out a list of 10 words and respondents were asked to repeat the words. | 0-10 |
|  | Delayed word recall | Respondents were asked to recall the same words read out for immediate  recall after some time. | 0-10 |
|  | Total word recall | Sum of immediate and delayed word recall | 0-20 |
| **Orientation** | Time | Respondents were asked to state today’s date, month and year and day of the week. For each question, the score was 0 or 1. Correct responses received 1point, incorrect responses received 0. The total score for time was 0-4. | 0-4 |
|  | Place | Orientation towards place was captured based on place of interview, name of the village, street number/colony name/landmark/neighbourhood and name of the district. Each correct response scored 1 point. The total score ranged from 0-4. | 0-4 |
| **Arithmetic function** | Backward counting | Respondents were asked to count backward as quickly as possible from the number 20. The respondents were asked to stop after correctly counting backward from 20 to 11 or from 19 to 10. Correct counting received 2 points; counts with a mistake received 1 point. Those who could not count received 0 points. | 0-2 |
|  | Serial 7 | Respondents were asked to subtract seven from 100 in the first step and asked to continue subtracting seven from the previous number in each subsequent step for five times. Each correct response received 1 point. | 0-5 |
|  | Computation | This test involved the mathematical operation of division. Respondents were asked to compute the net sale price of a product after considering a discount sale of half of the original price. | 0-2 |
| **Executive function: 0-4** | Executive (paper folding) | This is a three-stage command task. The respondents were instructed to take a piece of paper from the interviewer, turn it over, fold it in half, and give it back to the interviewer. Three points were given if each task was completed successfully. | 0-3 |
|  | Pentagon drawing | Visio-construction is the ability to coordinate fine motor skills with visuospatial abilities, usually by reproducing geometric figures. Respondents were asked to copy two overlapping pentagons and scored 1 point for a correct drawing. | 0-1 |
| **Object naming: 0-2** |  | The interviewer points to a specific object and asks the respondent to name it. Two objects were pointed out and 1 point was given for each correct response. | 0-2 |
| **Cognition** | Composite cognitive index | Combined score of memory (total word recall), orientation, arithmetic function, executive function, and object naming. | 0-43 |
