# Supplementary material for: Cognitive impairment and reporting of hypertension among adults in india: Evidence from a population-based study
Source: PLOS Ment Health. 2025 May 12;2(5):e0000003. doi: 10.1371/journal.pmen.0000003 (PMC12798364; doi:10.1371/journal.pmen.0000003)
Supplement: Attachment — Submitted filename: Response to Reiviewers_CN.docx [file pmen.0000003.s003.docx]

**Rebuttal Letter**

The authors extend their gratitude to the Editor and reviewers for their valuable feedback, which has greatly enhanced the manuscript.

**Reviewer’s comments with reply**

1. In line 59, too little fruit and vegetables does not explain diet-induced hypertension. The relevant sentence can be corrected as "poor eating behaviors". I think it is not correct to attribute hypertension only to fruit and vegetable consumption in terms of nutrition.

**Reply** – Eating too little fruits and vegetables is changed as “poor eating behaviours”

“According to specific epidemiological data, hypertension is also linked to behavioural risk factors such as smoking, drinking, insufficient physical activity, and poor eating behaviours^3-5^.”

2. I can say that the Introduction is not written in a fluent and explanatory way. Hypertension was determined as the universe of the subject, information about hypertension was given first, but the connection was not established in the transition from paragraph 1 to paragraph 2. However, it would be more appropriate to bring the subject to self-report in the 1st paragraph and then mention that cognitive impairment may negatively affect this.

**Reply** – considering the review, both paragraphs one and two are changed including the importance of self-reports and mentioning the role cognitive impairment has to play in it.

**“**Hypertension accounts for 19% of all non-communicable disease (NCD) deaths worldwide^1^. According to the World Health Organization (WHO), out of the total income loss of INR 1094-1113 billion arising from NCDs in India, a loss of INR 199 billion was on account of hypertension^2^ alone. Numerous population-based studies from low-income countries have shown that various socio-demographic characteristics are associated with hypertension, with higher rates of the disease being consistently associated with older age, female sex, and lower levels of education^3,4^. According to specific epidemiological data, hypertension is also linked to behavioural risk factors such as smoking, drinking, insufficient physical activity, and poor eating behaviours^3-5^. The data provided by large-scale surveys on the prevalence of hypertension is usually based on self-reports and standard tests. The inherent bias in the self-reporting of illnesses^6-8^ contributes to inaccuracies in disease reporting, which are further exacerbated by conditions such as cognitive impairment.

Cognitive impairment occurs due to the normal ageing process for various reasons and can affect the quality of life and even the ability to live independently^5,9,10^. It often affects elderly persons, adding to their functional disabilities and creating dependence, while also posing challenges in adapting to the changes in health technology. Meanwhile, young onset cases are also increasingly becoming common^11-14^. Evidence from the developed and developing nations suggests that age-related morbidities, functional limitations, poor mental health, and low quality of life among older adults are all associated with poor cognitive health^10,12,15–17^. Older adults suffering from cognitive impairment may experience memory loss and face issues with understanding, concentrating, and decision-making, all of which can lead to the false reporting of diseases, including hypertension, during the collection of data on self-reported morbidity^18,19^.”

3. I think it is not appropriate to talk about Alzheimer's and dementia because the research does not try to define these diseases.

**Reply** – The sentence describing Alzheimer's and dementia is removed from the manuscript.

4. The Cognitive paragraph in the Introduction section should be rewritten in a way that is linked to the previous paragraph and in line with the purpose of the research.

**Reply** – It is changed accordingly.

5. I think that the table names and table layouts are not in accordance with the journal's spelling rules.

**Reply** – Table layout and names are changed accordingly.

6. Paragraph 1 of the discussion section contains information that should be included in the introduction. It would be more accurate to prioritize the main findings of the research and explain the value of these data to the literature.

**Reply** – changes are made by avoiding 2 sentences in the first paragraph. Other sentences which are considered relevant for the discussion are kept as it is.

7. Discussion 323. Line; “Cognitive impairment is one of the most untreated ailments in India when compared to developed countries, and it is one of the most critical public health challenges. If left undetected in the elderly, it may progress to Dementia or Alzheimer.” This is not the purpose of the study; it is inappropriate to mention about it.

**Reply** – These sentences are removed from the manuscript considering the suggestions.

8. In line 359-362 “This variation between the odds ratios of self-reported and measured hypertension shows that cognitive impairment plays an important role in hypertension reporting, which might also be true in the case of other chronic conditions.” I think it is not correct to use such definitive sentences in the discussion section. Due to the nature of the study, it would be more accurate to make definitions such as "may be".

**Reply** – Changes are made accordingly, and a new paragraph is added which says “Further research is warranted to gain a better understanding and provide more clarity on this matter.”
