# Supplementary material for: Cognitive impairment and reporting of hypertension among adults in india: Evidence from a population-based study
Source: PLOS Ment Health. 2025 May 12;2(5):e0000003. doi: 10.1371/journal.pmen.0000003 (PMC12798364; doi:10.1371/journal.pmen.0000003)
Supplement: Attachment — Submitted filename: Reviewers Response_4_12_2024.docx [file pmen.0000003.s004.docx]

**Reviewers Response**

We would like to thank to Editor and both the Reviewers for their valuable suggestions to improve our manuscript.

Reviewer #2: 1. The abbreviation LASI in the keywords section should be written.

**Explanation: We have written the abbreviation LASI in the keywords section.**

2. In line 96 ‘Previous research has examined the discrepancy between self-reported hypertension and objective evaluations of hypertension evaluations and between false-negative and false-positive reporting factors and found.’ references should be added to the sentence.

**Explanation: We have added Reference.**

**Previous research has examined the discrepancy between self-reported hypertension and objective evaluations of hypertension and between false-negative and false-positive reporting factors and found the prevalence of self-reported and measured hypertension among older adults to be 27.4% and 31.5%, respectively, and the prevalence of total hypertension (average SBP ≥ 140 mmHg or/and DBP ≥ 90 mmHg or current use of any antihypertensive medication) to be 42.3%^22^.**

**Bhatia M, Dixit P, Kumar M, Dwivedi LK. Validity of self-reported hypertension in India: Evidence from nationally representative survey of adult population over 45 years. *J Clin Hypertens*. Published online 2022. doi:10.1111/jch.14542**

3. It is said that there is an older adults group, but how were the age categories created in the characteristic features in table 1? Why are 45-65 and under 45 included?

**Explanation: Thank you for your valuable suggestions. In this paper, we have included the entire surveyed population of 72,250 adults aged 45 years and above, including spouses regardless of their age. Preliminary analysis shows that approximately 4% of adults under 45 years and around 7% of those aged 45–65 years exhibit cognitive impairment, which is not negligible. Therefore, we did not exclude these groups from the analysis.**

**As our paper is not focused only on older adults, we have replaced the term "older" with "adult population" throughout the paper, as per your valuable suggestion. Thanks again for your important observations.**
