# Supplementary material for: Cognitive impairment and reporting of hypertension among adults in india: Evidence from a population-based study
Source: PLOS Ment Health. 2025 May 12;2(5):e0000003. doi: 10.1371/journal.pmen.0000003 (PMC12798364; doi:10.1371/journal.pmen.0000003)
Supplement: Attachment — Submitted filename: reviewer_response .docx [file pmen.0000003.s005.docx]

**Manuscript ID:** [PMEN-D-24-00001R2]

**Title:** Cognitive Impairment and Reporting of Hypertension among Adults in India: Evidence from a Population-Based Study

Dear Dr. Avanti,

Thank you for giving the opportunity to revise and resubmit the manuscript. Your comments and those of the reviewer have been very helpful in guiding the revision. We have revised the manuscript thoroughly in accordance with your recommendations and those of the reviewer. We provided a detailed summary of revisions in the accompanying memo. We believe that the manuscript has improved significantly as a result of the review process, and we hope that our revised study is suitable for a potential publication in *PLOS One Mental Health*.

**Response to Reviewers**

Thank you for your thorough review of the manuscript. We have addressed your concerns and believe that the revised manuscript is stronger as a result. In case where we were unable to make the revision, you suggested, we have offered our reasoning for such a decision. We have summarized our changes below and used track changes to highlight the revisions within the manuscript.

**Reviewer 3**

**Dear Authors,
The manuscript is well written. However, some changes to get a better clarity of data is needed. Please see the attached file for more details.**

Thank you. We are encouraged to know that you found our study interesting.

Additional notes:

**Line 114: Why restrict only to hypertension? Why not include type 2 diabetes and other chronic medical conditions**

**Explanation:** In LASI, only hypertension has been measured at the time of survey and data for the diabetes and other chronic conditions have not been released yet. We estimated misreporting hypertension based on the self-reported and measured hypertension.

**Line 126: A more detailed description is needed for this variable as it is important. Why was the cutoff chosen as the 10th percentile, why not the 25th percentile? Do the results change if the cutoff is changed?**

**Explanation:** Thank you for this suggestion; we acknowledge the importance of selecting an appropriate cut-off. Accordingly, we have incorporated the reference. In the Longitudinal Ageing Study, the lowest 10th percentile is used as a proxy indicator for poor cognitive functioning (Pandav et al., 2002), and this reference is already cited in the manuscript. Based on this cut-off, 9.8% of individuals in our sample are classified as cognitively impaired. If we were to use the 25th percentile as the threshold instead, approximately 22% would fall under the category of cognitive impairment. However, we have adhered to the approach used in the Longitudinal Ageing Study, employing the lowest 10th percentile as the standard for identifying poor cognitive function.

Ref: Pandav, R., Fillenbaum, G., Ratcliff, G., Dodge, H., & Ganguli, M. (2002). Sensitivity and specificity of

cognitive and functional screening instruments for dementia: The Indo‐US Dementia Epidemiology

Study. Journal of the American Geriatrics Society, 50(3), 554-561.

More description of the cognitive impairment variable, along with characteristics of its individual domains is provided in S1 Table.

**Page 10: Please mention how was missing data handled?**

**Explanation:** This is a valuable suggestion. Thank you. we have mentioned the missing cases in the last paragraph of the Statistical Analysis section.

Among the 72,250 total respondents, 9574 of them had missing information on cognitive functions. These observations were dropped during the multivariable analysis, and the final sample for the analysis was 62,676 respondents. Also, 35, 46, and 285 of the off-support observations were dropped from model 1, model 2, and model 3, respectively during the propensity score matching analysis because proper matching was not found.

**Page 12: mention numerator and denominator**

**Explanation:** Thank you for the valuable question.

Numerator - total cases of cognitive impairment in the total population of India (6022 cases)

Denominator - Total Indian population (63443 cases) after dropping missing cases.

**Page 13: Please include characteristics of cog unimpaired as well**

**Explanation:** We appreciate this suggestion. We have incorporated the characteristics of cognitively unimpaired in the revised manuscript (Table 1).

*Thank you for taking the time to offer feedback on our work.*
